# The cost of implementing Vietnam's national plan of action for nutrition for 2017–2020

**DOI:** 10.3934/publichealth.2019.3.276

**Published:** 2019-08-20

**Authors:** Hoang Van Minh, Vu Quynh Mai, Tran Tuan Anh, Nguyen Thuy Duyen, Le Danh Tuyen, Truong Tuyet Mai, Huynh Nam Phuong, Thahira Shireen Mustafa, Friday Nwaigwe, Do Hong Phuong

**Affiliations:** 1 Hanoi University of Public Health, No 1A Duc Thang street, Duc Thang Ward, North Tu Liem district, Hanoi, Vietnam; 2 National Institute of Nutrition, Hanoi, Vietnam; 3 Scaling Up Nutrition (SUN) Movement, Switzerland; 4 UNICEF Viet Nam, Hanoi, Vietnam

**Keywords:** nutrition policy, Vietnam, cost allocation, developing countries, national health programs

## Abstract

**Background:**

There is an urgent need to carry out a costing exercise of the National Plan of Action for Nutrition (NPAN) 2017–2020 since the costing of nutrition-sensitive interventions was not entirely integrated and proved difficult to track the different sectors' contributions to the nutrition program.

**Objective:**

To estimate the required budget for the activities of the NPAN in 2017–2020.

**Methods:**

A standard ingredients approach activity-based costing was employed from the provider perspective.

**Results:**

The budget amount required for the NPAN activities in 2017, 2018, 2019 and 2020 would be US$ million 269.0; 310.5; 350.2 and 378.1, respectively. State budgets (especially from Ministry of Health) would be the main funding source for the NPAN. The budget required for implementing nutrition-sensitive interventions would be the largest share (more than 90%) while less than 10% are required for nutrition-specific interventions. The four interventions requiring the largest budget proportion (in 2020) included 1) Micronutrient supplementation (28.3%); 2) Breastfeeding & complementary feeding (21.9%); 3) Treatment of severe acute malnutrition (15.6%); and 4) Disease prevention and management (13.4%).

**Conclusions:**

Based on the data from Vietnam National Health account and the data on GDP of Vietnam, the total required budget for the Vietnam NPAN 2017 (USD millions 5,082) as shares of the State budget for health, total State (Government) budget, and GDP would be 5.29%, 0.49% and 0.14%, respectively. From the estimation, Vietnam represents the nutrition strategy which prioritized on nutrition-sensitive actions, similar to most of the SUN Movement member countries.

## 1. Introduction

For a country that encountered severe food insecurity and famines in the 1980s, Vietnam has made an impressive achievement in combating malnutrition in the past three decades. Underweight among children under 5 years of age has reduced by nearly 70% between 1985 and 2015 [1]. Meanwhile, stunting has decreased by almost 60% since 1985, is at 24.6% in 2015 [1]–[3]. The progress in the reduction of malnutrition in Vietnam has been highly recognized and acknowledged by international organizations [4],[5]. The political concerns to prioritize nutrition came with the launch of the National Plan of Action for Nutrition (NPAN) for 1995–2000. As a follow-up to the NPAN 1995–2000, the National Nutrition Strategies for 2001–2010 and 2011–2020 was developed as the official document guiding nutrition policy for the Government [3]. After more than twenty years of the implementation of the NPAN for 1995–2000 with comprehensive approaches, multi-sector cooperation and governmental guidance at different levels, the nutrition status of Vietnamese people in general and that of mothers and children under 5 years of age in particular has improved remarkably together with enhancement in the awareness of nutrition issues among Vietnamese people.

With the vision toward 2030, Vietnam is faced with a big threat to the health and development of its people due to the multiple forms of malnutrition, particularly among the small children. Vietnam has just achieved its 2015 target indicated in the National Nutrition Strategy [3] for stunting (24.6% compared to 26% of target) and underweight (14.1% compared to 15% of target) and is currently struggling to sustain the achievement. Since 2015 one in four Vietnamese children under five still has experienced stunting (low height-for-age) and 6.4% of them are wasting (low weight-for-age) [6]. The number of severe acute malnourished children is about 200,000 annually [6]. The inequity has been raised significantly as more than 50% of the ethnic minority children living with multi-dimension poverty and not in sufficient nourishment [7].

Along with children malnutrition, the micronutrient deficiencies have been alerted when the prevalence of zinc deficiencies is 80% and 70% among women and children under five, respectively [8]. Regardless of the national program on Vitamin A supplement for under-five children, the prevalence of low serum vitamin A among them is still 13% [8]. Anemic is also common in Vietnamese women of childbearing age and in pregnancy (32.8%) and under-five children (27.8%) [8].

Beside the task of tackling the malnutrition problem, consideration efforts on overweight and obesity are also needed, otherwise burden of disease from diabetes and cardiovascular diseases would significantly increase. For people in middle-ages (45–54 years), overweight/obesity and metabolic syndrome are common, especially for urban citizens (43%, 17%, respectively) [9]. Non-communicable diseases go together with the double malnutrition burden. Prevalence of high blood pressure has increased from 1% in 1960 to 16.9% in 2001 [10] and to 20.3% in 2015 [10],[11]. Diabetes prevalence is about 5% but high elasticity according to differences region's nutrition diets [12]. On the other hand, nutrition issues are also influenced by the inadequacy of the management of systems governing agricultural products, food safety, water and sanitation [10]. The situation is more alerted in rural areas where still 21% of people here do not access to clean water services, 28% of them do not access to basic sanitation; 18% of them do not have handwashing point at home and 7.3% of them use open defecation in 2015 [7]. Funding cuts from the government started in 2015 and declining donor support threatened the sustainability of existing nutrition programs and the introduction of new initiatives [13]. In early 2015, a 65% decline in the public budget for nutrition was imposed by the government, which placed a great concern of maintaining the current coverage of nutrition services. In addition, the transition from low income to low middle-income status of the country has led to major changes in the aid structure and donor assistance in Vietnam. The Government of Australia decided to reduce 40% of its bilateral funding to Vietnam in May 2015 [14]. In March 2016, the United Kingdom's Department for International Development announced to phase out bilateral support to Vietnam [15]. Other sources of foreign funding have also been cut, leaving a significant reduction in the overall budget. Furthermore, international donors have changed their emphasis from funding traditional development and social programs to other fields such as governance and trade [16]. Investing in evidence-based nutrition interventions is important for improving children's health globally.

In 2014, the Government of Vietnam joined the Scaling Up Nutrition (SUN) Movement with a voluntary commitment to prioritize nutrition activities as part of their national agenda. As of 2018, sixty countries have committed to the Movement that recognizes the need to address all forms of malnutrition and its multiple causes. Costing and tracking of investment in nutrition plays a vital role in the policy planning, implementation, and monitoring cycle to prioritize resources and efforts for the forthcoming phase (2017–2020) in Vietnam. Therefore, working together closely across sectors to fully integrate nutrition programming into all development efforts is required. This needs close cooperation and support across ministries, and with donors, United Nations agencies, civil society groups and business enterprises. This will be increased even further by strong political leadership at the highest level to improve the nutritional status of the population.

In the NPAN 2011–2015, the costing of nutrition-sensitive interventions was not entirely integrated and consequently, it proved difficult to track the contributions of other sectors to the nutrition programs. Therefore, there is an urgent need to carry out a costing exercise of the NPAN, 2017–2020, which includes nutrition-specific, nutrition-sensitive, and governance actions.

Cost estimation should provide the required budget for the Government of Vietnam to achieve its nutrition targets set forth in the NPAN, 2017–2020. The specific objective of this research was to estimate the required budget for the activities of the NPAN, 2017–2020.

## 2. Methods

### 2.1. Design

A standard ingredients approach activity-based costing was employed. The costing was based on the “standard” resources needed to implement a set of “standard” activities within the NPAN 2017–2020. The “standard” activities and resources were jointly developed, discussed and agreed upon by all involved stakeholders. The activity-based costing (ABC) method defines the principal activities needed to implement the NPAN 2017–2020 and the ingredients approach identifies the types of inputs, the quantities of the inputs used by the program, and the cost per unit of the inputs. Only direct input costs are used in calculating the total program costs.

### 2.2. Cost perspective

The provider perspective was applied (the costs incurred by different ministries, institutions, and organizations).

### 2.3. Scope of the costing

Activities of both start-up (preparation or establishment) and running (maintenance) periods that are incurred by ministries, institutions, and organizations at all levels (central, provincial and local levels) were included. Start-up costs are resources used for activities implemented during the time between the decisions to implement a programme and starting its delivery to the first beneficiary (new activities). Implementation costs are resources used for routine activities.

Both capital and recurrent cost items were identified. Capital expenditures are the costs of acquiring fixed assets. Fixed assets are those assets, which are expensive, and give economic benefit to the health care provider for more than one accounting/financial year such as building, furniture, equipment, and vehicles. Recurrent costs are consumed within one financial or accounting year such as personnel, supplies, operation expenses including water, and electricity. Financial costs (accounting costs represent actual expenditures on goods and services purchased) are estimated.

### 2.4. Approach

We applied a standard ingredients approach to activity-based costing to estimate the cost of nutrition-specific activities within the scope of NPAN 2017–2020. Ingredients cover recurrent costs (personnel, intervention material, general expense, travel, advocacy/communication) and capital costs (building and equipment). To fill in the gap of knowledge in nutrition-sensitive activities, relevant cost information is taken from the expenditure review using a weighted 3-Step approach that includes identification, categorization and weighting of the allocated budget to nutrition relevant line items [17].

### 2.5. Study sites

All the organizations involved in implementing the NPAN 2017-2020, including National Institute of Nutrition, Ministry of Labour, War Invalids and Social Affairs, Ministry of Education and Training, Ministry of Agriculture and Rural Development, United Nations Children's Fund (UNICEF), Alive & Thrive, Global Alliance for Improved Nutrition (GAIN), Save the Children, United Nations Development Programme, Health Bridge, Vietnam World Vision, and Hellen Keller International.

### 2.6. Data collection

The research team worked together with the National Institute of Nutrition, UNICEF, and other relevant institutions and organization to:

Develop, discuss and agree upon the activities included in the NPAN 2017–2020. The draft version of NPAN 2017–2020 was used for this purpose.
Determine the standard procedure for implementation of each activity.
Identify types and quantity of resources needed to implement each activity.
Refine the list of activities, standard for implementation of each activity, types and quantity of resources needed to implement each activity by conducting interviews with nutrition experts at both national and local levels. Interviews with nutrition experts at local levels were done through field visits to three provinces (North, Centre and South).

### 2.7. Analysis

We estimated the costs for each activity identified in the NPAN, which will be implemented for the period of 2017–2020. We estimated costs according to groups of priority programs and for the groups of nutrition-specific (infant and young child feeding, micronutrient supplementation, nutrition interventions for communicable diseases, nutrition counseling, integrated management of acute malnutrition; control of overweight, obesity, and non-communicable diseases) and nutrition-sensitive (nutrition education, food security, food safety, water and sanitation, family planning, malaria prevention, deworming) activities.

### 2.8. Ethical approval of research

This study does not contain any procedure with human participants or animal performed by any of the authors. Informed consent was obtained from all individual participants included in the study.

The ethical approval for research was obtained from the Ethical Review Board for Biomedical Research of Hanoi University of Public Health.

## 3. Results

### 3.1. Required budget for activities of the National Plan of Action for Nutrition, 2017–2020

**Figure 1.**
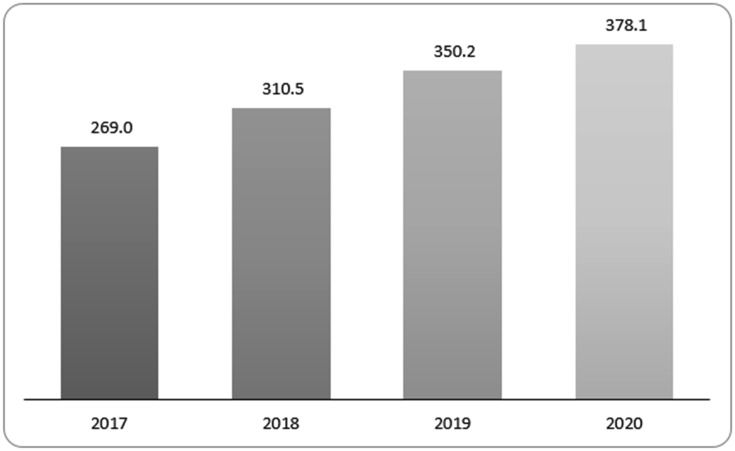
Required budget for Vietnam NPAN 2017–2020 (2017 Million US$).

Figure 1 shows the required budget (2017 US$) for the NPAN during 2017–2020. The budget amount required for the NPAN activities in 2017, 2018, 2019 and 2020 would be US$ million 269.0; 310.5; 350.2 and 378.1, respectively.

Table 1 presents the required budget for Vietnam NPAN 2017–2020 by source of funds. State budgets (especially the Ministry of Health budget) would be the main source of funds for the NPAN. Contributions from United Nations agencies, international non-governmental organizations, and non-governmental organizations would account for less than 15% of the total required budget.

**Table 1.** Required budget for Vietnam NPAN 2017–2020 by source of funds (2017 US$).

Source of fund	2017		2018		2019		2020		Total	
	Amount Million USD, %		Amount Million USD, %		Amount Million USD, %		Amount Million USD, %		Amount Million USD, %	
**State Budget**	**267.4**	**99.4%**	**307.7**	**99.1%**	**346.3**	**98.9%**	**373.4**	**98.8%**	**1,294.8**	**99.0%**
MOH	5.6	2.1%	8.3	2.7%	11.0	3.1%	12.9	3.4%	37.8	2.9%
Other Ministries	257.0	95.5%	291.7	93.9%	324.8	92.7%	347.6	91.9%	1,221.1	93.4%
**Local budget**	4.7	1.8%	7.6	2.5%	10.5	3.0%	13.0	3.4%	35.9	2.7%
Non-Government Organization	**1.7**	**0.6%**	**2.9**	**0.9%**	**3.9**	**1.1%**	**4.6**	**1.2%**	**13.0**	**1.0%**
UNDP	0.2	0.1%	0.3	0.1%	0.3	0.1%	0.4	0.1%	1.1	0.1%
NGOs	1.5	0.6%	2.6	0.8%	3.6	1.0%	4.3	1.1%	12.0	0.9%
Total	**269.0**	100%	**310.5**	100%	**350.2**	100%	**378.1**	100%	**1,307.8**	100%

Table 2 indicates the required budget for Vietnam NPAN 2017–2020 by NPAN objectives. The largest proportion of the budget would be for improving the quantity and quality of people's meals and the nutrition status of mothers and children (more than 30%).

**Table 2.** Required budget for Vietnam NPAN 2017–2020 by NPAN objectives (2017 US$).

NPAN Objective	2017		2018		2019		2020		Total	
	Amount Million USD, %		Amount Million USD, %		Amount Million USD, %		Amount Million USD, %		Amount Million USD, %	
1. Enhance policy formulation and interdisciplinary collaboration to promote & support nutrition activities	1.5	0.6%	2.1	0.7%	2.8	0.8%	3.0	0.8%	**9.4**	0.7%
2. People's meals are improved in terms of quantity and quality.	91.3	33.9%	103.3	33.3%	111.4	31.8%	119.3	31.6%	**425.3**	32.5%
3. Maternal and child nutrition status is improved.	77.8	28.9%	91.5	29.5%	111.8	31.9%	121.2	32.1%	**402.3**	30.8%
4. Micronutrient status is improved	76.7	28.5%	88.2	28.4%	95.8	27.3%	103.2	27.3%	**363.8**	27.8%
5. To effectively control overweight and obesity and risk factors of a number of nutrition related chronic NCDs in adults	21.6	8.0%	25.3	8.1%	28.0	8.0%	30.7	8.1%	**105.6**	8.1%
6. Monitoring, surveillance and evaluation	0.1	0.0%	0.2	0.1%	0.5	0.1%	0.6	0.2%	**1.4**	0.1%
Total	**269.0**	**100%**	**310.5**	**100%**	**350.2**	**100%**	**378.1**	**100%**	**1,307.8**	**100%**

Table 3 reports the required budget for Vietnam NPAN 2017–2020 by type of intervention. The budget required for implementing nutrition-sensitive interventions would make up the largest share (more than 90%). The share required for carrying out nutrition-specific interventions would account for up to 10% of the budget.

**Table 3.** Required budget for Vietnam NPAN 2017–2020 by type of intervention (2017 US$).

Type of intervention	2017		2018		2019		2020		Total	
	Amount Million USD, %		Amount Million USD, %		Amount Million USD, %		Amount Million USD, %		Amount Million USD, %	
**Nutrition-specific**	**12.5**	**5%**	**20.3**	**7%**	**27.3**	**7.8%**	**32.8**	**8.7%**	**93.0**	**7%**
MOH	4.8	1.8%	7.4	2.4%	9.8	2.8%	11.7	3.1%	33.7	2.6%
Other Ministries	1.5	0.6%	2.8	0.9%	3.7	1.1%	4.3	1.1%	12.3	0.9%
Local budget	4.6	1.7%	7.4	2.4%	10.0	2.9%	12.4	3.3%	34.5	2.6%
UNDP	0.1	0.0%	0.2	0.1%	0.2	0.1%	0.3	0.1%	0.9	0.1%
NGOs	1.5	0.6%	2.5	0.8%	3.4	1.0%	4.2	1.1%	11.6	0.9%
**Nutrition-sensitive**	**255.5**	**95.0%**	**288.8**	**93.0%**	**317.8**	**90.8%**	**340.0**	**89.9%**	**1,202.1**	**92%**
MOH	0.0	0.0%	0.0	0.0%	0.0	0.0%	0.0	0.0%	0.1	0.0%
Other Ministries	255.5	95.0%	288.8	93.0%	317.8	90.7%	340.0	89.9%	1,202.0	91.9%
Local budget	0.0	0.0%	0.0	0.0%	0.0	0.0%	0.0	0.0%	0.0	0.0%
UNDP	0.0	0.0%	0.0	0.0%	0.0	0.0%	0.0	0.0%	0.0	0.0%
NGOs	0.0	0.0%	0.0	0.0%	0.0	0.0%	0.0	0.0%	0.0	0.0%
**Environment enabling**	**1.0**	**0.38%**	**1.4**	**0.45%**	**5.1**	**1.47%**	**5.2**	**1.37%**	**12.8**	**0.98%**
MOH	0.7	0.3%	1.0	0.3%	1.2	0.3%	1.2	0.3%	4.1	0.3%
Other Ministries	0.1	0.0%	0.1	0.0%	3.3	0.9%	3.3	0.9%	6.7	0.52%
Local budget	0.2	0.1%	0.2	0.1%	0.5	0.1%	0.5	0.1%	1.4	0.1%
UNDP	0.0	0.011%	0.1	0.019%	0.1	0.019%	0.1	0.02%	0.2	0.02%
NGOs	0.0	0.0%	0.1	0.0%	0.1	0.0%	0.1	0.0%	0.3	0.02%
Total	**269.0**	**100%**	**310.5**	**100%**	**350.2**	**100%**	**378.1**	**100%**	**1,307.8**	**100%**

Table 4 shows the required budget for nutrition-specific activities in the Vietnam NPAN 2017–2020. The interventions requiring the largest budget shares would be 1) Micronutrient supplementation (28.3% in 2020); 2) Breastfeeding & complementary feeding (21.9% in 2020); 3) Treatment of severe acute malnutrition (15.6% in 2020); and 4) Disease prevention and management (13.4% in 2020).

**Table 4.** Required budget for nutrition-specific activities of Vietnam NPAN 2017–2020 (2017 US$).

Nutrition-specific interventions	2017		2018		2019		2020		Total	
	Amount Million USD, %		Amount Million USD, %		Amount Million USD, %		Amount Million USD, %		Amount Million USD, %	
1. Children health & preconception nutrition	0.2	1.6%	0.2	1.0%	0.2	0.8%	0.2	0.7%	0.9	0.9%
2. Maternal dietary supplementation	0.5	3.7%	0.7	3.4%	0.8	2.9%	1.0	2.9%	2.9	3.1%
3. Micronutrient supplementation	3.0	23.7%	5.3	25.9%	7.9	29.0%	9.3	28.3%	25.4	27.4%
4. Breastfeeding & complementary feeding	2.9	22.9%	4.7	23.3%	5.8	21.3%	7.2	21.9%	20.6	22.2%
5. Dietary supplementation	1.4	10.8%	2.6	12.8%	3.4	12.5%	4.0	12.1%	11.4	12.2%
6. Dietary diversification	0.0	0.2%	0.1	0.5%	0.2	0.8%	0.2	0.7%	0.6	0.6%
7. Feeding behaviors and stimulation	0.6	5.2%	0.9	4.2%	1.0	3.8%	1.2	3.8%	3.8	4.1%
8. Treatment of severe acute malnutrition	2.6	20.4%	3.4	16.8%	4.3	15.7%	5.1	15.6%	15.4	16.5%
9. Disease prevention and management	1.4	11.2%	2.4	11.7%	3.4	12.6%	4.4	13.4%	11.6	12.5%
10. Nutrition interventions in emergencies	0.0	0.2%	0.1	0.3%	0.1	0.5%	0.2	0.5%	0.4	0.4%
Total	**12.5**	**100%**	**20.3**	**100%**	**27.3**	**100%**	**32.8**	**100%**	**93.0**	**100%**

Table 5 shows the required budget for nutrition-sensitive activities in the Vietnam NPAN 2017–2020.The required budget for agriculture- and food safety-related activities would make up the largest share (35%).

**Table 5.** Required budget for nutrition-sensitive activities in Vietnam NPAN 2017–2020 (2017 US$).

Nutrition-sensitive interventions	2017		2018		2019		2020		Total	
	Amount Million USD, %		Amount Million USD, %		Amount Million USD, %		Amount Million USD, %		Amount Million USD, %	
1. Agriculture & food safety	91.3	35.8%	103.2	36%	111.2	35%	119.1	35%	424.9	35%
2. Social safety nets	1.0	0.4%	1.1	0%	1.2	0%	1.2	0%	4.4	0%
3. Early child development	24.6	9.6%	27.8	10%	30.0	9%	32.1	9%	114.6	10%
4. Maternal & reproductively health	42.3	16.6%	47.8	17%	51.5	16%	55.2	16%	196.9	16%
5. Women empowerment	0.0	0.0%	0.0	0%	0.0	0%	0.0	0%	0.1	0%
6. Child protection	0.4	0.2%	0.4	0%	0.5	0%	0.5	0%	1.8	0%
7. Classroom education	0.0	0.0%	0.0	0%	6.8	2%	6.8	2%	13.6	1%
8. Water and sanitation	75.0	29.3%	84.8	29%	91.3	29%	97.8	29%	348.8	29%
9. Other infectious & NCDs	20.8	8.2%	23.6	8%	25.4	8%	27.2	8%	96.9	8%
Total	**255.5**	**100%**	**288.8**	**100%**	**317.8**	**100%**	**340.0**	**100%**	**1,202.1**	**100%**

## 4. Discussion

Based on the data from Vietnam National Health account (the total annual government expenditures on health was USD millions 5,082 and the total annual government general budget was USD millions 54,348) [1] and the data on GDP of Vietnam (total annual GDP was USD millions 193,600) [2], the total required budget for the Vietnam NPAN 2017 (USD millions 5,082) as shares of the State budget for health, total State (Government) budget, and GDP would be 5.29%, 0.49% and 0.14%, respectively.

The required budget for the NPAN 2017 estimated from this study is quite similar to the figure of cost of nutrition packages in Vietnam estimated by Save Children in 2015 [18] (2015 US$ million 247, equal to 2017 US$ million 258). The budget estimated for 2017 from the current research (US$ million 536) is a bit higher than the figure estimated by World Bank in Vietnam 2010 (2010 US$ million 338, equal to 2017 US$ million 484.1) [19].

Table 6 shows the Government budget allocated to nutrition-specific and nutrition-sensitive interventions in 23 countries as a percentage of General Government Expenditures [19] as well as the figure for Vietnam estimated from this research. The figure for Vietnam of 0.49 % of General Government Expenditures was lower than that for all other countries investigated, except Zambia (0.14%).

**Table 6.** Government budget allocated to nutrition-specific and nutrition-sensitive interventions in 2016 for 24 countries as a percentage of General Government Expenditures.

No	Country	Percentage (%)
1	Guatemala	9.23
2	Comoros	5.11
3	Tajikistan	5.01
4	Peru	4.64
5	Nepal	3.59
6	Bangladesh	3.31
7	Costa Rica	3.01
8	Mauritania	2.11
9	Chad	1.49
10	Benin	1.48
11	Yemen	1.36
12	Ghana	1.21
13	Madagascar	1.21
14	Burundi	1.16
15	Pakistan	1.06
16	The Gambia	1.06
17	Burkina Faso	0.89
18	South Sudan	0.82
19	Kenya	0.70
20	Democratic Republic of the Congo	0.63
21	Indonesia	0.60
22	Philippines	0.52
***23***	***Vietnam***	***0.49***
24	Zambia	0.14

Source: Vietnam's percentage was calculated in this research. The remaining estimates are reported by The SUN Movement Secretariat, 2016 [20].

The nutrition plans at country-level vary depending on the country's context since there is no standard for investigating national budgets that could be applied by all. Vietnam represents the nutrition strategy which prioritized on nutrition-sensitive actions, similar to most of the SUN Movement member countries such as Bangladesh and Indonesia. The budget tracking of 24 SUN Movement member countries showed that most of the domestic budget allocations to nutrition identified by the countries relate to nutrition-sensitive intervention (1.7% of general government expenditures for sensitive and 0.4% for specific [21]).

In terms of nutrition-specific interventions, Vietnam spends 7.0% of the public expenditure and is 4th among countries with the lowest spending on nutrition-specific intervention. Comparing with other Asian countries in the SUN Movement, the figures of Vietnam are slightly higher than that of Indonesia (4.3%) and Bangladesh (3.6%) and much lower than Nepal (40.1%) [22]. Regardless of allocating 27.8% of the nutrition-specific budget to improve micronutrient status, the micronutrient deficiencies are still a persistent nutrition problem for Vietnamese. This implies a need for more concerns in improving both quality of the intervention programs and funding for the related programs.

For nutrition-sensitive interventions, with 92.0% government nutrition expenses, Vietnam's nutrition priority is similar to Bangladesh and Indonesia with the spending of 92.8% and 89.7% of national nutrition budget, respectively [22]. Among the nutrition-sensitive objectives, programs related to water & sanitation and maternal health also take place in the Sustainable Development Goals and several actual actions and multiplied-sources of funding have been allocated to these two objectives. Our study also found that the budget for nutrition-sensitive has come from the most at water and sanitation (29%) and maternal & reproductively health (16%) for the period of 2017–2020. The root of this phenomenon is the awareness and acceptance of the society for the need for development in water & sanitation and maternal health. Hence, related nutrition stakeholders (example, national nutrition department, the SUN, etc) might spend more efforts to raise nutrition awareness for other society-stakeholders. Nutrition-sensitive programs hold great promise for supporting nutrition improvements and boosting the scale, coverage, and benefits of nutrition-specific actions. Investments in nutrition-sensitive programs can have a pivotal role in the reduction of stunting, wasting, and impaired child development that the scale-up of nutrition-specific interventions cannot resolve on its own.

In summary, investing in ending malnutrition is one of the most cost-effective steps governments can take in which every $1 invested in proven nutrition programs offers benefits worth $16 [23]. However, nutrition has multiple underlying determinants and the solution to tackling malnutrition double burden is to using the public budget in the cost-effective way with a careful budget planning from the beginnings. Nutrition intervention should not just focus on meeting the demand of food production or a few typical proven nutrition-specific interventions but the concentration sould be paid for the multisectoral cooperation which could enabling better environment for nutrition status of general population. Investing in nutrition sensitive sectors such as agriculture, health and social protection; water, sanitation and hygiene as well as gender equity can help address 80% of stunting [24]. Government and non-government organizations in these sectors should recognise that nutrition is a collective responsibility, ear mark investments for nutrition, and work cohesively [25]. At the same time, governments, civil society organizations, donors, and other stakeholders should commit to ensure the expenditure for nutrition activities in various sector: agriculture, education, food systems, health systems, social protection, and water, sanitation, and hygiene which means to end all forms of malnutrition. We need more spending to build capacity to address obesity, diabetes, and other nutrition-related NCDs. And we need to start seeing nutrition investments as a means to economic growth rather than seeing better nutrition as a result of economic growth.

## 5. Limitations

This is the first estimation of the expenditure and required budget for the NPAN, 2017–2020. There were several limitations in the recent research:1) The estimates are likely under-estimated due to the unavailability of expenditure data from implementers of NPAN-related activities, especially agencies/institutions/organizations outside the health sectors; 2) The data received from the agencies/institutions/organizations outside the health sectors (for nutrition-sensitive activities) are in aggregate forms and lack details for the cost analysis. A weighting approach was applied as guided by the SUN Movement Secretariat, but this approach gives only broad cost estimates; 3) As the agencies/institutions/organizations outside the health sectors could not provide their detailed information on activities and associated costs required for nutrition-sensitive activities during 2017–2020, using the figures for 2015 from the expenditure analysis as rough estimates; 4) Only direct recurrent costs are included when estimating nutrition-specific activities. Opportunity costs (such as time costs) are not calculated. Non-recurrent direct costs (such as losses due to fire, theft; repair costs after natural disasters) are not included. Start-up costs for new activities (including hiring, guideline development) are also not included. Indirect costs (such as overhead and administrative costs not directly related to programme activities are also not included).

## References

[1] Alive&Thrive, National Institute of Nutrition, UNICEF (2013). Nationwide Nutrition Profile 2013.

[2] Ministry of Health (2001). National Strategy on Nutrition in 2001–2010 (Chien luoc dinh duong quoc gia ve dinh duong 2001–2010-Vietnamese version).

[3] Ministry of Health (2011). National Nutrition Strategy for 2011–2020 with a vision toward 2030.

[4] RESULTS UK, Concern Worldwide, University of Westminster (2015). What Works for Nutrition? Stories of success from Vietnam, Uganda and Kenya.

[5] Ministry of Planning and Investment (Socialist Republic of Viet Nam) (2013). Millennium Development Goals Full Report 2013: Achievement and challenges in the progress of reaching millennium development goals of Vietnam Hanoi, Vietnam: New Technology Printing Joint Stock Company.

[6] Nutrition NIf (2015). Nutrition Profile.

[7] UNICEF (2015). Children & Sustainable development goals-A snapshot: SDGs and Children in Vietnam.

[8] Ministry of Health (2015). National Micronutrient Survey in Vietnam.

[9] Khan NC, Khoi HH (2008). Double burden of malnutrition: The Vietnamese perspective. Asia Pac J Clin Nutr.

[10] PG K (2002). Situation of high pressure in Vietnam.

[11] Ministry of Health (2015). National Survey on the risk factors of Non-communicable diseases (STEPS) Vietnam 2015.

[12] TV B (2002). Epidemiology of diabetes, risk factors and related issues of diabetes management in urban areas of four major cities.

[13] International Food Policy Research Institute (2015). Global Nutrition Report 2015: Actions and Accountability to Advance Nutrition and Substainable Development.

[14] Australia Government-Department of Foreign Affairs and Trade (2015). Evaluation of the Australia-Vietnam country strategy 2010–2015.

[15] Development UG-DfI (2014). Operational Plan 2011–2016 DFID Vietnam.

[16] Tom Berliner, Do Kim Thanh, Adam McCarty (2013). Inequality, poverty reduction and the Middle Income Trap in Vietnam. Mekong Economics.

[17] Fracassi P, Picanyol C (2016). Tracking Government Investments for Nutrition at Country Level v.2. Scaling Up Nutrition.

[18] Save the Children (2010). Hungry for Change An eight-step, costed plan of action to tackle global child hunger Save the Children UK.

[19] Horton S, Shekar M, McDonald C (2010). Scaling Up Nutrition: What Will It Cost?. The World Bank.

[20] The SUN Movement Secretariat (2016). The scaling up Nutrition (SUN) Movement–Annual Progress Report 2016.

[21] Fracassi P, Picanyol C (2015). SUN Budget Analysis Synthesis Document: Establishing Nutrition Spending in SUN countries.

[22] Planning and costing for the acceleration of actions of nutrition: Experiences of countries in the Movement for Scaling Up Nutrition, 2014.

[23] International Food Policy Research Institute (2016). Global Nutrition Report 2016: From Promise to Impact: Ending Malnutrition by 2030.

[24] Bhutta ZA, Das JK, Rizvi A (2013). Evidence-based interventions for improvement of maternal and child nutrition: what can be done and at what cost?. The Lancet.

[25] Scaling Up Nutrition (2014). Effectively Engaging Multiple Stakeholders.

